# Curcumin for Treating Breast Cancer: A Review of Molecular Mechanisms, Combinations with Anticancer Drugs, and Nanosystems

**DOI:** 10.3390/pharmaceutics16010079

**Published:** 2024-01-05

**Authors:** Jing Zhu, Qian Li, Zhongping Wu, Ying Xu, Rilei Jiang

**Affiliations:** 1 School of Traditional Chinese Medicine, Shanghai University of Traditional Chinese Medicine, Shanghai 201203, China; zhujing0616@shutcm.edu.cn (J.Z.); wzp@shutcm.edu.cn (Z.W.); 2 Medical Department, Shanghai First Maternity and Infant Hospital, School of Medicine, Tongji University, Shanghai 200092, China; liqianv@tongji.edu.cn

**Keywords:** breast cancer, curcumin, drug delivery, nanosystems

## Abstract

Breast cancer (BC) has become the fifth most prevalent cause of cancer-related morbidity, attracting significant attention from researchers due to its heightened malignancy and drug resistance. Conventional chemotherapy approaches have proven inadequate in addressing all BC subtypes, highlighting the urgent need for novel therapeutic approaches or drugs. Curcumin (CUR), a phytochemical derived from Curcuma longa (turmeric), has shown substantial potential in inhibiting BC cell migration, metastasis, and proliferation. However, the use of CUR in this context comes with challenges due to its dynamic and easily degradable nature, poor aqueous solubility, low bioavailability, rapid metabolism, and swift systemic elimination, collectively limiting its clinical applications. As such, we provide an overview of the properties, synthesis, and characterization of the hybridization of CUR and its analogue with chemo-drug building blocks. We reviewed research from the last five years on CUR’s biogenesis with respect to the regulation of BC, revealing that CUR participates in arresting BC cells in the cell cycle and significantly induces apoptosis in BC cells. Information on the chemotherapeutic and antitumor mechanisms of CUR in BC, including regulation of the cell cycle, increased cell apoptosis, and inhibition of multidrug resistance (MDR), was compiled. Additionally, we provide an overview of CUR loaded into nanomaterials that are cotreated with other chemotherapeutic drugs, such as paclitaxel, thymoquinone, and tamoxifen. In this review, we discuss different types of nanoparticles that can be used for CUR delivery, such as polymeric nanoparticles, carbon nanotubes, and liposomes. By comparing the size, entrapment efficiency, drug-loading capacity, release time, biocompatibility, pharmaceutical scale, and reproducibility of various nanomaterials, we aimed to determine which formulations are better suited for loading CUR or its analogue. Ultimately, this review is expected to offer inspiring ideas, promising strategies, and potential pathways for developing advanced anti-BC strategy nanosystems in clinical practice.

## 1. Introduction

The incidence of breast cancer (BC) has been steadily increasing, surpassing that of lung cancer, making it currently the fifth leading cause of cancer-related morbidity [1]. BC is associated with four significant biomarkers: estrogen receptor (ER), progesterone receptor (PR), human epidermal growth factor 2 receptor (HER2), and Ki67. BC can be categorized into four subtypes based on the expression of these biomarkers: luminal A (ER/PR+, HER2−, Ki67−), luminal B (ER/PR+, HER2+, Ki67+), HER2-overexpressed (HER2+), and triple-negative breast cancer (TNBC) (ER−, PR−, HER2−) [2]. ER-positive BC cases account for approximately 70% of all BC cases, while TNBC, the most heterogeneous subtype, represents only 15% of BC cases [3]. Current effective therapies for BC encompass surgery, radiotherapy, chemotherapy, and hormonal therapy [4]. Nevertheless, studies have revealed drug resistance in traditional treatments, particularly among BC cell lines, with a specific emphasis on the TNBC subtype [5,6]. Consequently, the exploration of phytochemicals derived from herbs such as curcumin has piqued the interest of scientists as a potential component to enhance BC therapeutic strategies.

**Figure 1:**
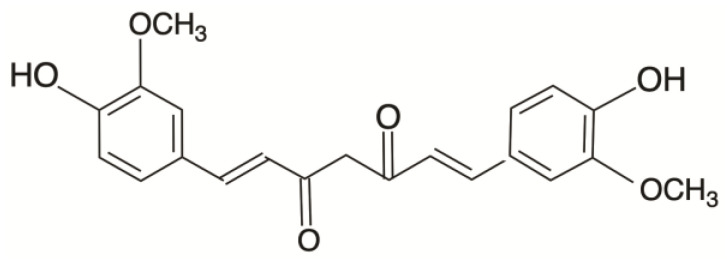
Chemical structure of curcumin (CUR).

Curcumin (C_21_H_20_O_6_) constitutes the primary bioactive compound found in the plant Curcuma longa, commonly known as turmeric. The chemical structure of curcumin (CUR) is visually depicted in Figure 1, comprising two phenyl rings adorned with hydroxyl (-OH) and methoxy (-OCH3) groups. Hydroxyl groups consist of an oxygen and a hydrogen atom, while methoxy groups consist of an oxygen atom and three hydrogen atoms. These functional groups can affect the chemical properties and reactivity of a compound. Notably, the hydroxyl radical (•OH) ranks as one of the most reactive and transient reactive oxygen species (ROS) produced in aerobic organisms [7], and its involvement was implicated in the induction of ferroptosis [8]. Combining curcumin with autophagy inhibitors (3-methyladenine) has been shown to trigger apoptosis and autophagy in castration-resistant prostate cancer cells. This combination also enhances the expression levels of TfR1 and IRP1, which are indicative of curcumin-induced iron deprivation [9]. In a separate study, Liu et al. suggested that platelets can inhibit the growth of methicillin-resistant Staphylococcus aureus by promoting excessive production of hydroxyl radicals, leading to induced apoptosis [10]. Wu’s research demonstrated that hydroxyl radicals can promote the generation of ROS, activate MAPK phosphorylation, and induce apoptosis in chemo-drug-resistant cells [11]. Additionally, curcumin has been found to regulate cancer cell proliferation by causing cell-cycle arrest at the G2/M phase [12].

From a molecular perspective, previous meta-analyses reinforced the ability of curcumin to reduce CRP (C-reactive protein) concentrations [13]. CUR possesses a wide range of pharmacological activities and has been extensively studied for new drug development. Furthermore, the coadministration of CUR with other phytochemicals improves anticancer activity by regulating multiple molecular targets [14] and modulating various intracellular signaling pathways, including transcription factors such as NF-κB [15], Akt [16], CDK, MAPK [17], ERK [18], and Bcl-2 [19]. In traditional Chinese medicine, curcumin was employed in clinical meta-analyses for the treatment of conditions such as arthritis [20], pain and analgesia [21], menstrual pattern disorders [22], and premenstrual syndrome [23]. 

Over time, CUR and its analogue have been extensively validated for their relevance to cancer treatments [24,25]. CUR has been employed as an adjuvant in various cancer types, including lung cancer, brain cancer, and BC [26,27], maximizing the pharmacokinetic profile, ensuring specific cell internalization, and enhancing anticancer efficacy against BC [28]. Hence, CUR was investigated as a potential anti-BC agent that disrupts a positive loop through the CXCL12/CXCR4 axis [29].

## 2. Bioavailability of Curcumin

Curcumin, a natural compound found in the turmeric plant Curcuma longa, is generally considered safe when consumed in amounts commonly found in foods and traditional herbal remedies. In 2021, the European Food Safety Authority (EFSA) established an acceptable daily intake of CUR at 3 mg/kg body weight [30]. For optimal pharmacological effects, an oral dose of more than 8.0 g/day is often required. Numerous clinical studies demonstrated that a daily intake of 12 g of CUR is well tolerated and safe [31]. 

One challenge with curcumin is its low solubility in water. Curcumin is hydrophobic, meaning it does not readily dissolve in water. This characteristic makes it difficult for the body to absorb when ingested. Curcumin is primarily found in its keto form under acidic and neutral conditions [32] and is highly unstable. Studies show that approximately 90% of curcumin degrades within 30 min when exposed to a pH of 7.2 at 37 °C in a 0.1 M phosphate buffer and serum-free medium [33].

The efficiency of CUR metabolism varies among species, including mice, rats, and humans, whereby metabolic-O-conjugation to curcumin-O-glucuronide (COG) and curcumin-O-sulfate (COS) and bioreduction to tetrahydrocurcumin (THC), hexahydrocurcumin (HHC), and octahydrocurcumin (OHC) rapidly occur in the liver and intestines, further limiting its bioavailability [34]. Research by Vareed et al. revealed that only small amounts of unaltered CUR remain in the body’s systemic circulation [35]. The compounds can also be broken down and metabolized in an acidic environment, leading to gastrointestinal degradation.

Given curcumin’s limited bioavailability, low water solubility (≤0.125 mg/L), rapid metabolism, and quick elimination from the body, it is considered an unstable, reactive, and poorly bioavailable compound [23], resulting in restricted clinical use [24]. Therefore, the main emphasis of this review is to present an overview of the clinical applications of CUR in BC. This includes its use in combination with other chemotherapy drugs and in nanobased systems to improve the bioavailability of CUR.

## 3. Curcumin Biogenesis in the Regulation of Breast Cancer

Curcumin has been utilized for centuries in traditional medicine, particularly in traditional Chinese medicine herbal systems. Previous studies demonstrated that curcumin possesses potential chemical properties contributing to anti-breast cancer (anti-BC) effects on several phenotypes through the following mechanisms: (a) inhibition of P-glycoprotein activity and reduction in drug resistance; (b) induction of the cell cycle; (c) initiation of apoptosis and ferroptosis; and (d) regulation of the progression of the epithelial–mesenchymal transition (EMT). These mechanisms were validated in both in vivo and in vitro studies. In this section, we focus solely on reviewing how curcumin influences changes in the phenotype of breast cancer rather than its combination with traditional chemotherapy drugs.

### 3.1. CUR Inhibits p-Glycoprotein Activity and Reduces Drug Resistance in BC

In the late 1970s and early 1980s, Drs. Victor Ling and I. David Goldman first proposed the concept of P-glycoproteins (P-gp) and the characterization of P-gp as a membrane protein associated with resistance to chemotherapy in cancer cells. The overexpression of P-gp can expel most chemotherapeutic agents, significantly contributing to P-gp-mediated multidrug resistance (MDR) and leading to the intracellular accumulation of antitumor drugs [29]. In this section, we exclusively review how CUR affects changes in MDR rather than its combination with traditional chemotherapy drugs.

CUR has been illustrated as a P-gp inhibitor which can prevent BC-MDR progression. The CUR metabolism product tetrahydrocurcumin can inhibit the efflux function of P-gp and extend the MDR-reversing activity of curcuminoids in vivo [36]. CUR itself was also demonstrated to be a novel inhibitor of P-pg, enhancing the response to traditional chemotherapy drugs. Attia et al. demonstrated that combined CUR and D3 could enhance tumor response to PAX and inhibit aldehyde dehydrogenase-1 (ALDH-1) and P-gp-MDR levels [37]. Curcumin analogue was proven to reduce MDR1 protein expression and reverse P-gp-MDR to enhance sensitivity to PAX [38]. CUR inhibits the efflux function of P-pg transfecting the protein ABCB4, which is reverse-doxorubicin-resistant in BC cell lines [39]. 

### 3.2. Curcumin Induces Cell-Cycle Arrest in BC Cells

The cell cycle is an ordered set of events that leads to cell growth and division (G1, S, and G2 phases), and it protects proliferating cells from DNA damage [40]. CUR and CUR analogues have been suggested to amplify the protective functions of BC cells in arresting the cell cycle.

While every phase is involved in cancer progression, the G1 phase is often considered particularly crucial in promoting cancer progression due to its initial position and function, whereby cells duplicate themselves. CUR-loaded solid lipid nanoparticles (SLNs) arrest the cell cycle at G1/S and decrease the expression of cyclin D1 (CCND1) and CDK4, which strongly induce apoptosis and ROS reactions [41]. The codelivery of salinomycin suggests that HA-PEG-PLGA-Cur-Sal is the most effective in preventing BC from subsequently progressing into the S phase, where genome duplication occurs [42]. The CUR analogue B14 induces G1 phase cell-cycle arrest and activates the mitochondrial apoptosis pathway by altering the expression of cyclin D1 (CCD1), cyclin E1, and cyclin-dependent kinase 2 (CDK2) [43].

**Figure 2:**
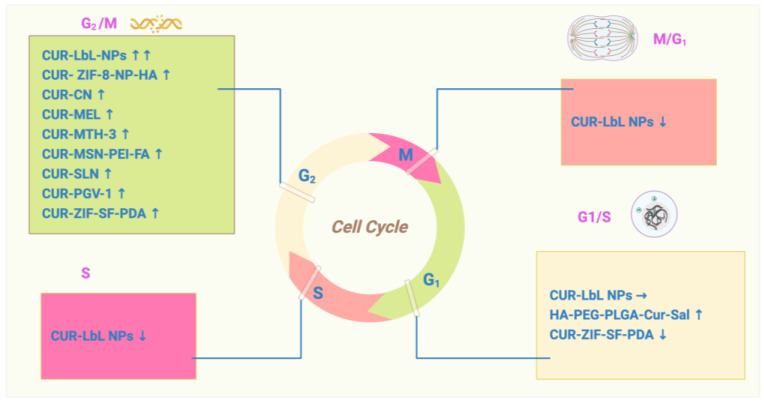
The role of curcumin in cell-cycle regulation: CUR induces cell-cycle arrest in BC cells. The cell cycle is an ordered set of events that leads to cell growth and division (G1, S, and G2 phases).

However, most CUR combinations arrest the cell cycle at the G2/M phase. The integration of CUR with layered polyelectrolyte capsule (LbL) nanotemplates (NPs) showed a significant increase in the number of cells at G2. Therefore, the percentage of apoptotic cells was significantly increased [44]. Mesoporous silica nanoparticles can affect the cell cycle by disrupting the microtubule assembly. Nana Li et al. compared the efficiency effects of CUR-MSN-HA and CUR-MSN-PEI-FA in MDA-MB-231 and MCF-7 cell lines, consequently proving that CUR-MSN-polyethyleneimine (PEI)-FA is more effective at inducing the G2/M phase cell-cycle arrest [45]. The CUR analogue (2E,6E)-2,6-bis-(4-hydroxy-3-methoxybenzylidene)-cyclohexanone (BHMC) was shown to promote G2/M cell-cycle arrest and apoptosis in MCF-7 cells [46]. CUR increased apoptosis in blocked MDA-MB-231 cells at the G2 phase and inhibited the growth of TNBC by silencing EZH2 and restoring DLC1 expression [47]. Intriguingly, some research suggests a possibility that traditional chemotherapy drug cotreatment with CUR showed different efficiencies in BC cells and normal epithelial cells. Wei Yang Kong et al. illustrated that CUR and doxorubicin (DOX) treatments induced G2/M arrest in MDA-MB-231 MCF-7 and MCF10A; however, CUR induced S phase arrest in MCF10A [48]. The CUR-related molecule pentagamavunon-1 (PGV-1) induces arrest at the M phase of the cell cycle and subsequent cell senescence and cell death [49]. Resveratrol (RSV) is a naturally occurring compound associated with a reduction in CCNB1, PLK1, AURKA, and AURKB along with an increase in CDKN1A (p21) [50]. Data show that resveratrol treatment results in a reduction in S phase cell cycle and induction of γ-H2AX [51] (Figure 2).

### 3.3. Curcumin Induces Apoptosis in BC Cells

Apoptosis is the process of programmed cell death. Alterations in apoptosis can lead to uncontrolled cell division and, consequently, tumor growth and resistance to antitumor therapies. The relationship between the cell cycle and apoptosis is undoubtedly a crucial aspect of cellular regulation and maintenance of tissue homeostasis. Curcumin-loaded micelles were previously shown to efficiently penetrate MCF7 spheroids and induce apoptosis [52].

On the one hand, CUR and its analogues activate ROS-related signaling pathways. The CUR analogue WZ35 inhibits cell growth via the ROS-YAP-JNK signaling pathway in BC [53]. CUR-ZIF-8 and CUR-ZIF-8-HA significantly elevate intracellular ROS levels, subsequently resulting in the dysfunction of mitochondria and apoptosis in 4T1 cells [54]. Several studies showed that CUR effectively induces apoptosis and autophagy in TNBC cell lines, for example, MDA-MB-231. Moreover, cotreatment with curcumin and the chemotherapeutic drug melphalan increased MDA-MB-231 ROS levels 1.36-fold and induced apoptosis [55].

**Figure 3:**
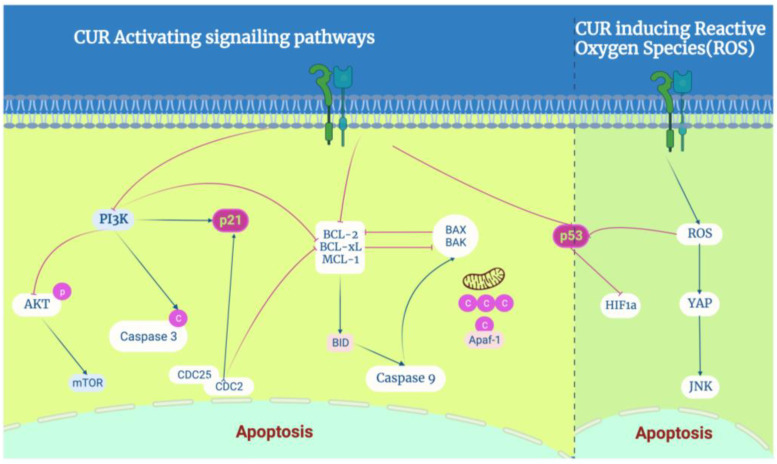
The role of curcumin in apoptosis regulation: CUR induces apoptosis in BC cells, especially by activating P53 and P21 proteins to regulate the molecular targets of curcumin in breast cancer, which activates the PI3K-AKT, NF-kB, P53, and p21 signaling pathways. On the other hand, it induces ROS-mediated activation of typical P53 and JNK signaling pathways.

On the other hand, CUR and its analogues were revealed to be significantly related to canonical apoptosis transactor proteins P53 and P21. The activates of p53 could enhance the transcription of proapoptotic Bcl-2 family members. The codelivery of CUR and Bcl-2 siRNA induced apoptosis [19], and the CUR analogue curcumin nicotinate (CN) inhibited cell growth and proliferation via p53-mediated cell-cycle arrest at the G2/M phase and apoptosis [56]. The activates of p21 typically inhibit the kinase activity of cyclin-dependent kinases (CDKs), including those associated with CCND1, CDK4, or CDK6, and induce cell-cycle arrest. Curcumin may affect the cell cycle by regulating the expression of the regulatory proteins CDC25 and CDC2 via the P21 inhibitor at the G2/M phase [57]. Data suggest that proteasome-mediated downregulation of cyclin E and upregulation of CDC inhibitors contribute to the antiproliferative effects of CUR [58]. A combination of CUR and thymoquinone against BC decreased caspase-3, phosphatidylinositol 3-kinase (PI3K), and protein kinase B (AKT) protein levels, which is strongly related to P21 [59] (Figure 3).

## 4. Hybridization of Curcumin and Chemotherapeutic Drug Delivery in Nanosystems

Multidrug resistance (MDR) in tumors is recognized as a significant risk factor for the failure of chemotherapy, accounting for approximately 90% of cancer-related deaths [49]. Some studies indicated that CUR is a potential ingredient that could be encapsulated with chemotherapeutic drugs to help reverse MDR in BC. Common chemotherapy drugs include the anthracycline DOX, alkylating agent cyclophosphamide, antimicrotubule agent taxeme, and antimetabolite 5-FU. Below, we report the synergism that exists between combinations of CUR and drugs such as PTX, thymoquinone, tamoxifen, and resveratrol (RSV) and their performance in nanodelivery systems.

### 4.1. Curcumin Encapsulated with Paclitaxel (PTX)

Multifunctional lipid nanoparticles are potential candidates for the codelivery of PTX and CUR for targeted delivery and enhanced cytotoxicity in multidrug-resistant breast cancer cells [60]. The augmentation of the therapeutic effectiveness of the coadministration novel cationic PEGylated niosomal formulations of paclitaxel and curcumin in an MCF-7 cell line was studied [61].

The attachment of the lipoid HA-HAD to the surface of hydrophobic PLGA nanoparticles to codeliver CUR and PTX can activate interactions between HA and CD44 receptor targets on the membrane of BC cancer stem cells [62]. Amphiphilic heparin-poloxamer P403 (HSP) nanogel that could load CUR and paclitaxel PTX encapsulation through the hydrophobic core of poloxamer P403 confirmed a lower cytotoxicity of HSP-CUR-PTX compared to free PTX as well as a higher inhibition effect with MCF-7 [63]. The self-assembly-engineered PTX-CUR nanodugs showed higher therapeutic efficiency and better prognosis than free PTX and the simple PTX-Cur mixture [64]. Biotin-poly(ethylene glycol)-poly(CUR-dithiodipropionic acid) (Biotin-PEG-PCDA) polymeric nanocarrier loaded with PTX, magnetic nanoparticles (MNPs), and quantum dots (QDs) was developed to overcome the drug resistance mechanisms of the MDR-MCF-7 model [65]. The prepared PTX-CUR-NPs had a smaller size with a low polydispersity index and showed a slow release of PTX and CUR without any burst effect [66]. PTX-CUR-SLN has been verified as a promising therapeutic nanoparticle-based therapy for BC and provides a novel strategy to solve the problems of low efficacy and poor safety of clinical chemotherapy drugs [67].

### 4.2. Curcumin Encapsulated with Tamoxifen (TAM)

Tamoxifen (TAM) is a drug usually selected for ER-positive BC cell patients. Its mechanism of action involves blocking hormones that stimulate tumor cell development [68]. However, its frequent use causes serious side effects, including endometrial cancer, polyps, secondary endometrial cancer, hyperplasia, and thromboembolic events. A previous study showed that CUR and tamoxifen decreased the viability of BC cell lines MCF-7/LCC2 and MCF-7/LCC9 and induced cell-cycle arrest at the G2/M phase [69]. Curcumin may prevent H19 metastasis in MCF-7/TAMR cell epithelial–mesenchymal transition-associated metastasis [70]. TAM-CUR-loaded niosomes caused the upregulation of bax and p53 genes and downregulation of Bcl2 owing to the higher cell uptake via the niosomal formulation in MCF-7 [71]. 

### 4.3. Curcumin Encapsulated with Doxorubicin (DOX)

Reversion of multidrug resistance was demonstrated via the coencapsulation of doxorubicin and curcumin in chitosanpoly(butyl cyanoacrylate) nanoparticles [72]. CUR-loaded solid lipid nanoparticles (SLNs) bypassed P-pg MDR in TNBC cells [73]. Amphiphilic copolymeric micelles were employed for doxorubicin and curcumin codelivery to reverse multidrug resistance in breast cancer [74].

Curcumin reverses doxorubicin resistance via inhibiting the efflux function of ABCB4 in doxorubicin-resistant breast cancer cells [39].

The (DOX-CUR) micelle-treated group exhibited the highest rate of ATP inhibition, indicating that its P-gp inhibition ability occurred through a decrease in energy availability. Compared with Dox treatment alone, the results showed that 15 µM of CUR combined with DOX significantly increased apoptosis in proliferative MCF7 cells [75]. A novel biocompatible magnetic nanomedicine based on beta-cyclodextrin, loaded with doxorubicin-curcumin, was evaluated for overcoming chemoresistance in breast cancer [76].

Table 1 presents the combined use of CUR with traditional chemotherapeutic drugs for the treatment of BC. Most assays indicated that the combination with CUR improved the inhibition of and treatment efficacy for BC.

### 4.4. Curcumin Encapsulated with Methotrexate (MTX)

Methotrexate is an antimetabolite and antifolate drug that inhibits the metabolism of folic acid by inhibiting the enzyme dihydrofolate reductase, therefore disrupting the synthesis of DNA, RNA, and proteins and affecting the rapid growth of BC cells. The codelivery of the model chemotherapeutic MTX and CUR was achieved by combining nanocrystalline cellulose (NCC) and the amino acid L-lysine for an efficient delivery to MCF-7 and MDA-MB-231 cells [77]. MTX and curcumin-coencapsulated PLGA nanoparticles upgraded the EE and LC of CUR, which seems to be a potential BC therapeutic strategy to treat BC [78]. Additionally, a nanocarrier system derived from the self-assembly of a dextran–curcumin conjugate prepared via enzyme chemistry, with immobilized laccase acting as a solid biocatalyst, was designed to effectively deliver MTX to BC cells [79].

### 4.5. Curcumin Encapsulated with Other Chemotherapy Drugs

5-Fluorouracil (5-FU), docetaxel (DTX), and other chemotherapy drugs were consistently combined with CUR and delivered in nanosystems to enhance efficacy. 5-FU is a pyrimidine analogue that interferes with the synthesis of DNA and RNA. CUR, berberine, and a combination with 5-FU loaded in nanomicellar particles were able to exert a more pronounced effect on MCF7 cells at lower doses [80]. CUR can be encapsulated with gemcitabine as a nanosuspension to enhance its anticancer potentiality synergistically [81]. The codelivery of CUR and the chemotherapeutic drug docetaxel for the treatment of BC addresses MDR and the better-sustained release effects [82]. CUR can be coadministered with docetaxel as a nanosuspension to enhance its anticancer effect by increasing its oral bioavailability and decreasing drug efflux [83] (Table 2).

**Table 1:** CUR cytotoxicity in combination with other chemotherapeutic drugs.

Drug/Chemotherapeutic	IC50	Cell Lines	Treatment Time	References
CUR-PTX-D3	10.94		168	[37]
CUR-TAM	9.815 10.93	MCF-7/LCC2 MCF-7/LCC9	24, 48	[69]
CUR-TAM	31.7	MCF-7/TAMR	48	[70]
CUR-GEM-NP	6.9 4.0 5.5	MCF-7 MDA-MB-231 B16F10	24	[81]

## 5. Delivery Platforms of Curcumin in Nanosystems

**Figure 4:**
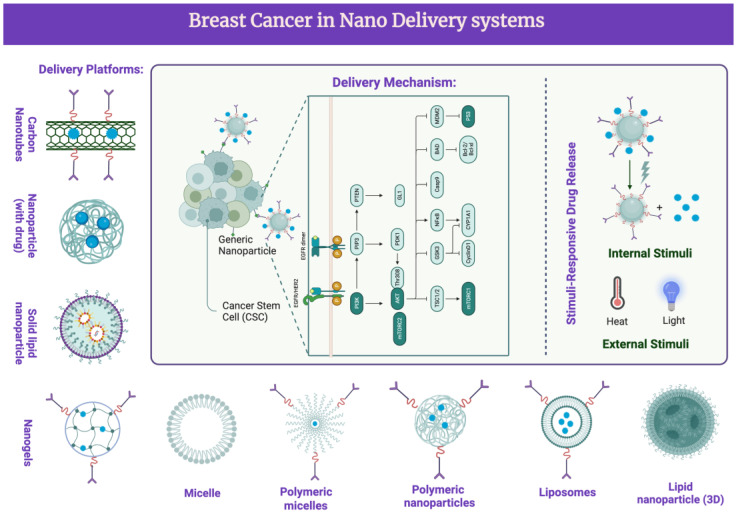
Nanodelivery systems in BC clinical treatments with CUR and its analogues. CUR and its analogues were delivered via different platforms. This activates several signaling pathways and target molecules. Phosphoinositide 3-kinases (PI3Ks); protein kinase B (Akt); B-cell lymphoma 2 (Bcl-2); epidermal growth factor receptor (EGFR); cyclin-dependent kinase inhibitor (CDK); vascular endothelial growth factor (VEGF); matrix metalloproteinases (MMP); extracellular signal-regulated kinases (ERKs); nuclear factor kappa-light chain enhancer of activated B cells (NF-κB); poly (ADP-ribose) polymerase (PARP); downregulated targets; upregulated targets.

Nanosystems typically range from 1 to 100 nanometers. They can be classified according to their composition, structure, and intended applications and include nanoparticles, nanocomposites, nanotubes, nanofibers, nanoliquids, and other nanoporous materials. Strategies such as nanotechnology have been used to load CUR in nanosystems to enhance its anti-BC effects. Upon comparing different capping agents, such as chitosan, dextran, and PEG and an emulsifier (tocopherol poly(ethylene glycol)1000, succinate, TPGS) to those of PLGA NPs, the efficiency of encapsulation with PLGA NPs was demonstrated to be much higher than any other cappers [84]. Herein, we provide an overview of CUR in different nanomaterials and their loading capacity and drug release time (Figure 4). This research illustrates that delivery in nanosystems highly improves the usage of CUR.

### 5.1. Polymers for the Delivery of Curcumin

PLGA (poly(lactic-co-glycolic acid)) is a copolymer of lactic acid and glycolic acid and has been approved by both the Food and Drug Administration (FDA) and European Medicines Agency (EMA) as a safe substance certified for clinical application. Curcumin-loaded PLGA NPs demonstrated high encapsulation efficiency and sustained payload release [85]. GANT61 and curcumin-loaded PLGA nanoparticles targeted GLI1 and PI3K/Akt-mediated inhibition in BC [86]. CUR encapsulated by poly(N-isopropylacrylamide-co-methacrylic acid) NIPAAm-MAA nanoparticles efficiently inhibited the growth of the MCF-7 population, providing potential for new avenues for BC treatment [87].

Different acids were used to modify nanoparticles to enhance the efficiency of CUR. Fangyuan Guo et al. demonstrated that DOX and CUR encapsulated by folic acid-modified nanoparticles based on a star-shaped polyester (FA-TRI-CL) strongly enhanced BC-targeting selectivity and drug-loading capacity compared to NPs without FA. This suggests that folic acid may be an innovative modification carrier [88]. Hyaluronic acid (HA) modification was carried out on the surface of curcumin nanocrystals (Cur-NCs) to obtain surface-reformed hydrophilic HA-Cur-NCs with a prolonged biodistribution in both MDA-MB-231 cells and a murine 4T1 orthotopic BC model, providing a prospect for promoting CUR absorption in vivo and in vitro [89]. Poly-glycerol-malic acid-dodecanedioic acid(PGMD) was fabricated to convey CUR in nanoparticles for the treatment of BC and showed apoptotic features via the overexpression of caspase 9 and induced nuclear anomalies in the treated MCF-7 and MDA-MB-231 cells [90]. A nanohybrid based on (Mn, Zn) ferrite nanoparticles functionalized with chitosan and sodium alginate was employed for the loading of CUR against BC cells [79].

Chitosan (Ch) is another popular material used for coating drugs. Ch-coated iron oxide nanoparticles (Ch-IONPs) were fabricated to deliver a prodrug of CUR, i.e., CUR-diethyl γ-aminobutyrate (CUR-2GE). In the presence of permanent magnets, CUR-2GE-Ch-IONPs significantly increased the cellular uptake of and cytotoxicity toward MDA-MB-231, with a 12-fold increase in potency compared to free CUR-2GE, indicating the potential of magnetic field-assisted delivery of CUR-2GE-Ch-IONPs for the treatment of triple-negative breast cancer [91]. Injectable and in situ-formable thiolated chitosan-coated liposomal hydrogels were studied as CUR carriers for the prevention of in vivo BC recurrence [92]. Curcumin-loaded solid lipid nanoparticles bypassed P-glycoprotein-mediated doxorubicin resistance in triple-negative breast cancer cells [73]. The codelivery of salinomycin and CUR for cancer stem cell treatment occurred via the inhibition of cell proliferation, cell-cycle arrest, and epithelial–mesenchymal transition [42] (Table 3).

### 5.2. Liposomal Formulations for the Delivery of Curcumin

Over the past decades, research efforts have been concentrated on liposomes, i.e., spherical vesicles characterized by a membrane comprising a double layer of phospholipids and cholesterol. Liposomes play a crucial role in safeguarding drugs from degradation and mitigating drug-associated nonspecific toxicity. Their primary applications encompass the transportation of antibiotics, fungicides, vaccines, and anti-inflammatory drugs [100]. Nanoscale liposomes are increasingly recognized as a highly beneficial drug delivery systems for anticancer agents, leading to an enhanced treatment of drug-resistant tumors and diminished toxicity [101]. CUR was encapsulated by RGD-modified liposomes (RGD-Lip-CUR) and induced apoptosis in MCF-7 cells, indicating high cytotoxicity effects on a BC cell line [98]. 

When HER2-targeted immunoliposomes are coupled with trastuzumab, there is a dramatic increase in the antiproliferative effects of CUR and an increase in the positive therapeutic effect [102]. Liposome-loaded metal ions (Zn) as nanoscaled reaction vehicles were used to carry out a synthesis reaction between Zn and CUR and presented enhanced cellular-uptake and ROS-generation effects [103]. Double-layer cisplatin (Cis)-membrane-intercalated CUR reduced cytotoxic effects and was capable of inducing apoptosis in BC cells [99].

### 5.3. Inorganic Nanomaterials for the Delivery of Curcumin

Inorganic nanomaterials are composed of non-carbon-based elements.

The codelivery of curcumin and letrozole in NiCoFe2O4-L-Silica-L-C-niosome enhanced the apoptosis rate in both MDA-MB-231 and SK-BR-3 cells and downregulated Bcl-2, MMP 2, MMP 9, and cyclin D [97]. CUR-loaded SLN enhanced anticancer efficiency in BC [41]. A type of composite nanoparticles loaded with epirubicin and CUR within an SLN system loaded for treating recurrent BC showed good blood and immune compatibility and did not affect intracellular superoxide dismutase (SOD) and intracellular catalase (CAT) [104]. An ingenious nonspherical mesoporous SLN cargo with CUR induced mitochondria-mediated apoptosis in breast cancer (MCF-7) cells [105]. Hydrazinocurcumin-encapsuled nanoparticles “re-educated” tumor-associated macrophages and exhibited antitumor effects on breast cancer following STAT3 suppression [106].

### 5.4. Polymeric Micelles for the Delivery of Curcumin

Nanogel-based drug delivery systems have been broadly used for cancer treatment.

CUR-octadecylamine (ODA1, OAD2, OAD3) showed that the presence of CUR-loaded chondroitin sulfate nanogels could successfully increase cellular uptake in comparison with free curcumin [93]. HA-decorated mixed nanomicelles loaded with CUR highly expressed CD44 receptor to provide an efficient drug delivery system [107]. The preparation of CUR TPP-PEG-PE nanomicelles with mitochondrial targeting and lysosomal escape functions exhibited the effect of promoting BC cell apoptosis [108]. Cholesterol- and vitamin E-conjugated PEGylated polymeric micelles were also employed for the efficient delivery and enhanced anticancer activity of curcumin [109]. 

### 5.5. Newly Developed Platforms for the Delivery of Curcumin

Recent years have witnessed a captivating surge in the discovery and exploration of innovative materials, capturing the attention of researchers and scientists globally.

Cell-permeable NBD peptide-modified liposomes encapsulated by hyaluronic acid coating were employed for the synergistic targeted therapy of metastatic inflammatory breast cancer [110]. 

Carbonic anhydrase IX-guided albumin nanoparticles were studied for hypoxia-mediation of triple-negative breast cancer cells in the killing and imaging of patient-derived tumors [111]. Curcumin–human serum albumin nanoparticles decorated with PDL1-binding peptide were used for targeting PDL1-expressing breast cancer cells [95]. Aptamer-functionalized curcumin-loaded human serum albumin (HSA) nanoparticles were employed for targeted delivery to HER2-positive breast cancer cells [108].

Silk fibroin (SF)-related nanoparticles show great potential in developing alternative carriers for anticancer drugs due to their biocompatibility and low immunogenicity. Edy Meiyanto et al. produced a core–shell microfluidic-assisted ZIF-8 nanoparticle protected by SF as an intermediate layer and coated by PDA for zinc ion release. It successfully reduced the nanoparticle size from 1000 nm to 170 nm, which enhanced permeability and retention (EPR) effects [94]. C. Laomeephol produced dimyristoyl glycerophosphorylglycerol (DMPG)-based liposomes for inducing the rapid gelation of SF and delivering CUR, which enhanced the stability of CUR [112].

Graphene oxide (GO) and graphene quantum dots (GQDs) are suitable nanocarriers for hydrophobic and low-bioaccessible antitumor materials such as CUR [113]. The synthesis and characterization of a CHIT–carbon quantum dot/Fe(2)O(3) nanocomposite comprising curcumin was used for targeted drug delivery in BC therapy [114]. Phenyl boronic acid (PBA)-conjugated ZnO nanoparticles were synthesized for the tumor tissue-specific delivery of curcumin and caused apoptotic cell death in MCF-7 human BC cells by inducing ROS and mitochondrial damage [115].

## 6. Discussion

Curcumin is a natural compound found in the rhizomes of turmeric, a member of the ginger family. It is a polyphenolic compound with a chemical structure that includes two aromatic rings and a diketone group. Many polyphenolic compounds, including CUR, act as antioxidants. Moreover, this review provides an overview not only of CUR but also of its analogue, which shows more hydrophilicity among the chemical properties. In Section 2, we reviewed research from the previous five years relating to CUR’s biogenesis in the regulation of BC. Studies show that CUR participates in arresting BC cells in the cell cycle and significantly induces the apoptosis of BC cells. Some research illustrates that CUR and its analogues are involved in the progression of ROS, which aligns with its chemical structure characteristics.

We compiled information on the chemotherapeutic and antitumor mechanisms of CUR in BC, including the regulation of the cell cycle, increased cell apoptosis, and inhibition of MDR. Additionally, we provided an overview of CUR loaded in nanomaterials cotreated with other chemotherapeutic drugs such as paclitaxel, thymoquinone, and tamoxifen. These combinations significantly improve antitumor activity and reduce toxicity via synergistic and additive effects against BC cells. Previous investigations validated the potential of CUR and chemotherapy drugs in new combinations for selectively targeting cancer cells and suppressing MDR. To date, the exact mechanisms underlying these chemotherapeutic and antitumor effects are not fully understood. However, its hydrophobic nature limits its clinical use.

Upon comparing the clinical treatment effects between CUR-combined chemotherapies with delivery via nanosystems or without such delivery, we concluded that the former is far more effective. Therefore, it is necessary to produce an effective formulation to load CUR or its analogues. Knowledge of the pharmacokinetics and pharmacodynamics of nanosystems also needs to be updated. For efficient clinical translation, a more rational design of nanoparticles loaded with CUR is necessary for preclinical experimentation and clinical trials against BC.

Currently, nanotechnology-based delivery systems are being investigated to minimize risks to humans and increase the chemotherapeutic effects of CUR. In this review, we discussed different types of nanoparticles that can be used for CUR delivery, including polymeric nanoparticles, carbon nanotubes, and liposomes. Comparing the size, entrapment efficiency, drug-loading capacity, release time, biocompatibility, pharmaceutical scale, and reproducibility of different nanomaterials further elevated the elucidation of the kind of formulations that are better for loading CUR or its analogues. Hyaluronic acid (HA) hydrophilic surface-rehabilitated CUR may be suitable to modify the formation, which can be combined with nanoparticles. From Table 2, we can conclude that HA-modified nanomaterials show higher entrapment efficiency and drug-loading capacity. PLGA is a common nanoparticle; however, whether it is the perfect choice for CUR is hard to determine. The key technical difficulty is to overcome CUR’s low water solubility by increasing the EE and LC of CUR and its analogues. According to the above tables, we can conclude that the narrower hydrate size showed better levels of EE and LC to enhance clinical treatments in BC. Rising materials, such as SF-related nanoparticles and GQD, reveal the significant potential of employing nanodelivery systems. Upon comparing graphene oxide with GQD, the latter showed clearly better clinical results in BC, mainly different due to its the material diameter.

In conclusion, CUR delivery via nanodelivery systems for treating BC is worth further investigating.

## 7. Conclusions

CUR unquestionably exhibits potential as an anticancer agent, with relevance not only to breast cancer but also to lung cancer, gastric cancer, and other malignancies. Previous studies indicated that CUR’s low delivery efficiency, attributed to its rapid metabolism and swift systemic elimination, can be enhanced via the combination of nanosystem delivery and nanotechnology.

While this review presents the prospect of delivering CUR via nanosystems for the treatment of breast cancer, it is imperative to recognize that there is still a considerable journey ahead in order to completely elucidate the intricate mechanisms of curcumin concerning various types of cancers.

## Figures and Tables

**Table 2:** CUR combined with chemotherapy drugs in nanosystems.

Drug/Chemotherapeutic Nanosystem	Cell Line	Hydrated Size (nm)	Zeta-Potential (mV)	Entrapment Efficiency	Drug-Loading Capacity	Release Time	References
CUR-PTX-HA-HAD	MCF7	200–400	−26.5	78.9 ± 5.5	23.8	12	[62]
CUR-TAM-niosomes	MCF7	159.45		98.37	20.68 ± 1.25	24	[71]
CUR-MTX-PLGA	SK-BR-3	148.3 ± 4.07	3.41 ± 0.8	71.32 ± 7.8	22.1 ± 2.85	72	[78]
CUR-MTX-NCC	MCF-7 MDA-MB-231	336.7	−33.1		22.44	48	[77]
CUR-DTX-Lip	MCF7	208.53 ± 6.82	−23.1 ± 2.1	98.32% ± 2.37%	59.27%	24	[82]

**Table 3:** Polymers for the Delivery of Curcumin.

Nanosystem	Hydrated Size (nm)	Zeta-Potential (mV)	Entrapment Efficiency	Drug-Loading Capacity	Release Time	References
CUR-CS-ODA-Nanogel	311 ± 20.29	−13.25 ± 0.35	79.56 ± 5.56%	6.55 ± 2.88%	70 h	[93]
CUR-ZIF8-NP-HA	170.6 ± 11.2	−18.10 ± 1.08	56.7%	10.1 ± 1%	10 h	[54]
CUR-HA-PEG-PLGA-PEG	153.4 ± 4.6	−32.6 ± 2.5			6 h	[42]
CUR-ZIF8-SF-PDA	196	−32 ± 30	44%	8.2%	24 h	[94]
CUR-PTX-SLN	238.5 ± 4.79	−33.8 ± 1.26	94.2 ± 0.49%	10.98 ± 0.31%	24 h	[67]
CUR-GANT61-PLGA-NPs	347.4 ± 2.75	−21.3 ± 0.23	98.3 ± 0.33% and 99.97 ± 0.09%	25.6 ± 1.23% and 28.6 ± 2.05%	24 h	[86]
CUR-NIPAAm-MAA	166 ± 6.0		89.6%		24 h	[87]
CUR-DOX-FA-NPs	186.53 ± 2.78	−18.87	97.64%	20.27%	24 h	[88]
CUR-peptide-HSA-NPs	246.5 ± 2.5	−24.5 ± 1.5	77.8%	5.52%	12 h	[95]
CUR-HA-NC	161.85 ± 1.70	−25.0 ± 0.8	80%		48 h	[89]
Cu-ALG-CHITr-MNP	122.4 ± 4.10	20–40	70%	35%	36 h	[96]
CUR-Letrozole NiCoFe2O4-L-Silica-L-C-Niosome	120.1		92.73%, 81.21%	28.7%	8 h	[97]
CUR-RGD-Lip	97.4 ± 7.10		76.86 ± 7.52%	28.96 ± 3%	3 h	[98]
CUR-DTX-Lip	208.53 ± 6.82	−23.1 ± 2.1	98.32 ± 2.37%	59.27%	24 h	[82]
CUR-PTX-NP	125.1 ± 0.44	−24.16 ± 0.22	54.12 ± 0.22%	28.16%	72 h	[61]
CUR-Cis-NLips	174.9 ± 18.4		99.81%	23.86%	24	[99]

## Data Availability

Not applicable.
